# CD133 antigen expression in ovarian cancer

**DOI:** 10.1186/1471-2407-9-221

**Published:** 2009-07-07

**Authors:** Gabriella Ferrandina, Enrica Martinelli, Marco Petrillo, Maria Grazia Prisco, Gianfranco Zannoni, Stefano Sioletic, Giovanni Scambia

**Affiliations:** 1 Gynecologic Oncology Unit, Department of Oncology, Catholic University, Campobasso, Italy; 2 Gynecologic Oncology Unit, Catholic University, Rome, Italy; 3 Institute of Human Pathology, Catholic University, Rome, Italy

## Abstract

**Background:**

Much attention has been recently focused on the role of cancer stem cells (CSCs) in the initiation and progression of solid malignancies. Since CSCs are able to proliferate and self-renew extensively, thus sustaining tumor growth, the identification of CSCs through their antigenic profile might have relevant clinical implications. In this context, CD133 antigen has proved to be a marker of tumor cells with stemness features in several human malignancies.

The aim of the study was to investigate the clinical role of the immunohistochemically assessed expression of CD133 in a large single Institution series of ovarian cancer patients.

**Methods:**

The study included 160 cases admitted to the Gynecologic Oncology Unit, Catholic University of Campobasso and Rome. CD133 antigen was identified by the monoclonal mouse anti-CD133-1 antibody (clone CD133 Miltenyi biotec).

**Results:**

In the overall series CD133 positive tumor cells were observed in 50/160 (31.2%) cases. A *diffuse cytoplasmic *pattern was identified in 30/50 (60.0%), while an *apical cytoplasmic *pattern was found in 20/50 (40.0%) of CD133 positive tumors.

As of September 2008, the median follow up was 37 months (range: 2–112). During the follow up period, progression and death of disease were observed in 123 (76.9%), and 88 (55.0%) cases, respectively. There was no difference in TTP between cases with negative (median TTP = 23 months) versus positive CD133 expression (median TTP = 24 months) (p value = 0.3). Similar results were obtained for OS. When considering the TTP and OS curves according to the pattern of CD133 expression, a trend to a worse prognosis for cases with *diffuse cytoplasmic *versus the *apical cytoplasmic *pattern was documented, although the statistical significance was not reached.

**Conclusion:**

The immunohistochemical assessment of CD133 expression seems not to provide additional prognostic information in ovarian cancer patients. The role of the different pattern of CD133 immunoreaction deserves further investigation in a larger series.

## Background

Increasing attention is currently focused on the role of cancer stem cells (CSCs) in the initiation and progression of leukemias and solid malignancies [1]. In particular, in the CSC model only a small proportion of tumor cells are proposed to be able to proliferate and self-renew extensively, thus sustaining tumor growth, while the bulk of cell populations proceed to differentiate into heterogeneous clones that become the phenotypic signature of the tumor [1]. The existence of CSCs was first demonstrated by transplantation of a small population of harvested leukaemia cells from patients into immunodeficient mice, which then developed the same malignancy [2]. Since then, CSCs have been described in several human tumors including breast, gastrointestinal, lung, prostate, brain, and melanoma on the basis of their clonogenic efficiency *in vitro *and ultimately tumorigenicity *in vivo *[3-9]. The identification and characterization of CSCs might have unpredictably huge clinical implications: for instance, it is has been shown that CSCs might survive chemo- as well as radiotherapy [10-14], due to the preferential expression of resistance molecules or activation of specific signaling pathways. It is therefore intuitively conceivable that only the eradication of CSCs or, alternatively, the induction of CSCs differentiation into cells lacking self-renewal potential can lead to an effective cancer cure. CSCs in specific tumor types are associated with elevated expression of the stem cell surface marker CD133 [15], which can be ultimately used for the identification as well as purification and enrichment of CSCs population [4-7], by means of a specific antibody directed against the AC133 and AC141 epitopes. CD133 (formely known as AC133) is a 5-transmembrane cell surface glycoprotein located in plasma membrane protrusions where it could act as a regulator of lipid composition, cell polarity and migration [16].

In transplantation experiments CD133^+ ^glioblastoma as well as colon cancer cells successfully induced tumors in immunodeficient mice [4-7]. Moreover, CD133 antigen expression has been identified in various types of solid tumours [5,9,10,16-18], and documented to be associated with patients' response to treatment and clinical outcome; in particular, CD133^+ ^glioma cells were shown to exhibit resistance to radiation [17], and chemotherapy [18]. Moreover, elevated blood levels of CD133 mRNA have been shown to be associated with poor overall survival in patients with bone metastatic disease [19], and with a higher risk of recurrence in colon cancer [20] Some authors have also addressed the clinical role of the immunohistochemically assessed CD133 expression in gliomas as well as pancreatic and hepatocellular carcinomas [21-24]: with the limits of some methodological differences (type of antibody, definition of the cut-off values of CD133 positivity, etc), the unfavourable prognostic role of high CD133 expression has been recognized in most of the studies [21-23]. As far as ovarian cancer is concerned, isolation and characterization of cellular clones with clonogenic potential *in vitro *and tumorigenicity *in vivo *even after serial transplantation in mice, have been recently documented [25]. We recently showed that that CD133 antigen represents a useful molecule in order to select and enrich the population of ovarian tumor cells expressing a higher clonogenic efficiency and proliferative potential [26]. Moreover, CD133^+ ^Ovarian cancer cell lines have been very recently shown to exhibit enhanced resistance to platinum-based therapy, and to form more aggressive tumor xenografts at a lower inoculum than their CD133^- ^progeny [27,28].

To the best of our knowledge, no data has been reported until now about the potential clinical role of the immunohistochemically assessed expression of CD133 and its in ovarian cancer in a large, single Institution series of primary untreated ovarian cancer patients.

## Methods

### Patient population

The study included 160 ovarian cancer patients admitted to the Gynecologic Oncology Unit, Catholic University of Campobasso and Rome between May 1999 and January 2006. Although this study only included a retrospective collection of archival data, the protocol was submitted to our Institutional Review Board, and approved for the use of patients' clinical information. Clinico-pathological characteristics of the overall series are summarized in Table 1. Forty-seven (29.4%) patients were >65 years old (median age = 58 years, range:24–83). One-hundred nineteen cases (74.4%) were stage III, and 21 (13.1%) cases were stage IV disease. Serous histotype was documented in the vast majority (n = 115, 71.9%) of cases. All patients underwent primary surgical effort at the Catholic University of Campobasso-Rome by only two surgical teams, which share the same surgical approach, and are able to obtain the same results in terms of percentage of optimal cytoreduction at primary surgery. Maximal surgical effort has been attempted in all patients resulting in optimal debulking (apparently absent residual tumor) in 52 (32.5%) cases, which underwent surgical removal of tumour masses, along with total abdominal hysterectomy, adnexectomy, radical omentectomy, appendectomy, multiple biopsies, and additional surgery (intestinal resections, diaphragm stripping) when required.

**Table 1:** Clinico-pathological characteristics of the overall series, and percentage of cases with positive CD133 expression

Characteristics	No. pts (%)	No. (%) of cases with positive CD133 expression	**p value**^**a**^
**All cases**	160	50 (31.2)	

**Age (yrs)**			
<65	113 (70.6)	36 (31.9)	
≥ 65	47 (29.4)	14 (29.8)	0.8

**FIGO Stage**			
I-II	20 (12.5)	6 (30.0)	
III	119 (74.4)	39 (32.8)	
IV	21 (13.1)	5 (23.8)	0.7

**Grade**			
G1-2	28 (17.4)	7 (25.0)	
G3	107 (66.8)	34 (31.8)	0.5
n.a.	35 (21.8)		

**Histotype**			
Serous	115 (71.9)	33 (28.7)	
Other	45 (28.1)	17 (40.5)	0.2

**Residual tumor**			
Absent	52 (32.5)	19 (36.5)	
Suboptimal	40 (25.0)	8 (20.0)	
Exploratory laparotomy	68 (42.5)	23 (33.8)	0.2

**Response to treatment**			
Yes	130 (81.2)	43 (33.1)	
No	30 (18.8)	7 (23.3)	0.3

^a^calculated by Fisher's exact test for proportion or X2 test

n.a. = not available

Suboptimal cytoreduction was achieved in 40 (25.0%) cases, while 68 cases (42.5%) were judged to be unresectable at first surgery because of extensive peritoneal bulky carcinomatosis, agglutinated bowel/mesentery and infiltration of the upper gastrointestinal tract and/or the major vessels, and were submitted only to multiple biopsies. All patients received platinum-based chemotherapy (75–100 mg/m^2 ^for cisplatin, AUC = 5 for carboplatin, per cycle), plus paclitaxel (135–175 mg/m^2 ^for each cycle). As far as patients undergoing only exploratory laparotomy are concerned, they received 3–4 cycles of chemotherapy before attempting a second cytoreductive surgery, unless they showed clinical progression during treatment. In all patients response to chemotherapy was assessed according to WHO criteria [29]. For the purposes of data analysis, complete and partial response were grouped together and classifies as "response to treatment", while cases with stabilization of disease or progression were considered as "no response to treatment".

### Immunohistochemistry

Pre-treatment tumor tissues biopsies were obtained at first surgery in all cases. Immunostaining was performed as previously described [26]. Briefly, 3 μm formalin fixed, paraffin-embedded slides from cancer tissue sections, were deparaffinized in xylene and rehydrated conventionally; the endogenous peroxidase was blocked with 3% H_2_O_2 _for 5 min. To reduce non specific binding the sections were incubated with 20% normal goat serum for 30 min, at room temperature. Cells expressing CD133 antigen were identified after overnight incubation at 4°C by using the monoclonal mouse anti-CD133-1 antibody (clone CD133 Miltenyi biotec) (1:50 dilution) which has been shown to provide the most sensitive results with the lowest background, and has been previously used to identify cells with stemness features in other human malignancies [24,30]. CD133 detection was performed by using anti-mouse EnVision System-HRP (DakoCytomation, Carpinteria, CA, USA), for 30 min. at room temperature. Diaminobenzidine was used as a chromogen (DAB substrate System, DAKO). Immunohistochemical stained slides were reviewed by two investigators (GZ, EM) independent from one another, and blinded to clinical data. CD133 stained cells were counted in 10 random and non-overlapping fields at high magnification (400×), and the results were expressed as the percentage of total number of nuclei counted in the same fields [22]. For analysis of survival patients were divided into negative (% CD133^+ ^tumor cells = 0) versus positive (% CD133^- ^tumor cells>0) CD133 expression.

### Statistical analysis

X2 test or Fisher's exact test for proportion were used to analyze the distribution of cases with positive CD133 expression according to clinico-pathological parameters. Time to progression (TTP) and overall survival (OS) were calculated from the date of diagnosis to the date of progression/death or date last seen. Medians and life tables were computed using the product-limit estimate by the Kaplan and Meier method [31], and the log-rank test was employed to assess the statistical significance [32]. Statistical analysis was carried out using SOLO (BMDP Statistical Software, Los Angeles, CA). Multivariate analysis assessing the clinical role of CD133 expression matched with other clinico-pathological characteristics was performed by Cox's proportional hazards model [33].

## Results

### CD133 expression protein in primary ovarian cancer

**Figure 1:**
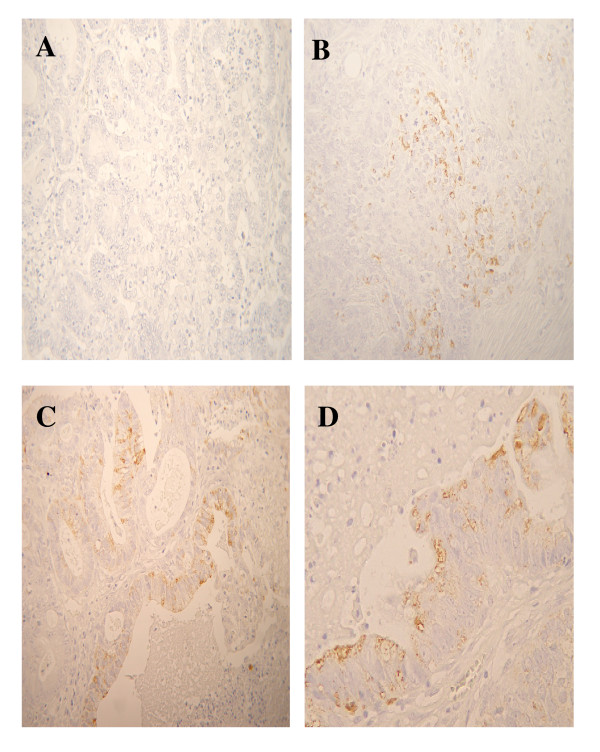
**CD133 immunoreaction in primary ovarian cancer**. A) Negative control; B) Representative example of CD133 positive immunoreaction in the cytoplasm of solidly arranged tumor cells; C, D) Representative examples of CD133 positive staining at the apical/endoluminal portion of tumor cells. Magnification = 100× (A, B, C); 200× (D).

Figure 1 shows representative examples of CD133 antigen expression in primary ovarian cancer: Immunoreaction appeared to be present as a diffuse staining of cytoplasm (*diffuse cytoplasmic *pattern) of tumor cells forming solidly arranged tumors or at the apical/endoluminal surface (*apical cytoplasmic *pattern) of tumor cells surrounding a lumen.

**Figure 2:**
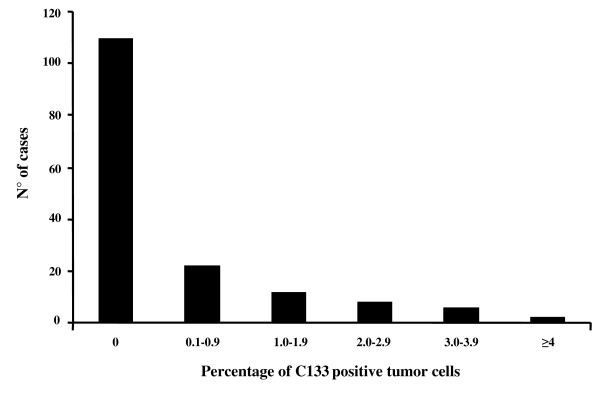
**Distribution of the values of the percentage of CD133 positively immunostained tumor cells**.

The distribution of the percentages of CD133 stained cells is shown in Figure 2: in the overall series CD133 positively stained tumor cells were observed in 50/160 (31.2%) cases, and the mean +SE values of proportion of CD133 positive cells was = 0.48 +0.08 (range:0–4.93). Moreover, the *diffuse cytoplasmic *pattern was identified in 30/50 (60.0%), while the *apical cytoplasmic *pattern was found in 20/50 (40.0%) of CD133 positive tumors. There was no difference in the distribution of cases with positive CD133 expression according to any of the clinico-pathological parameters examined, including response to chemotherapy (Table 1).

As of September 2008, the median follow up was 37 months (range: 2–112). During the follow up period, progression and death of disease were observed in 123 (76.9%), and 88 (55.0%) cases, respectively. Given the intertumor variability, the absence of a defined scoring system, and the need to minimize any source of bias related to the use of a specific cut off value, the possible clinical role of CD133 expression in ovarian cancer was examined by Cox proportional hazard model using the percentage of Cd133 positive tumor cells as a continuous value, and by Kaplan and Meier analysis.

No association between the percentage values of positively CD133 positive immunostained tumor cells expressed as continuous value and the relative risk of progression (X^2 ^= 2.96, p value = 0.09), or death of disease (X^2 ^= 0.10, p value = 0.75) was found (data not shown).

**Figure 3:**
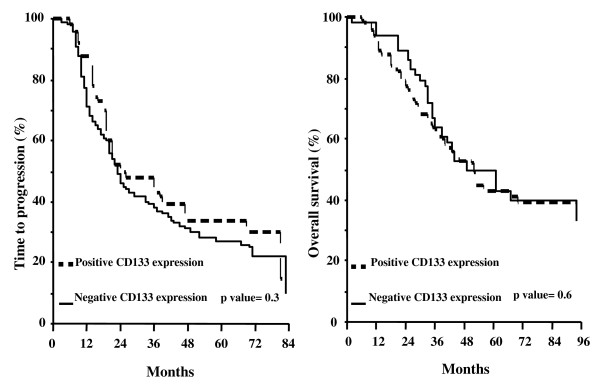
**Time to progression (A) and overall survival (B) curves in ovarian cancer patients according to the status of CD133 expression**.

Figure 3 shows the TTP and OS curves according to CD133 expression status: there was no difference in TTP between cases with negative (median TTP = 23 months) versus positive CD133 expression (median TTP = 24 months) (p value = 0.3). Similar results were obtained for OS: cases with negative CD133 expression experience a median OS of 52 months versus a median OS of 49 months in positive CD133 expression (p value = 0.6).

**Figure 4:**
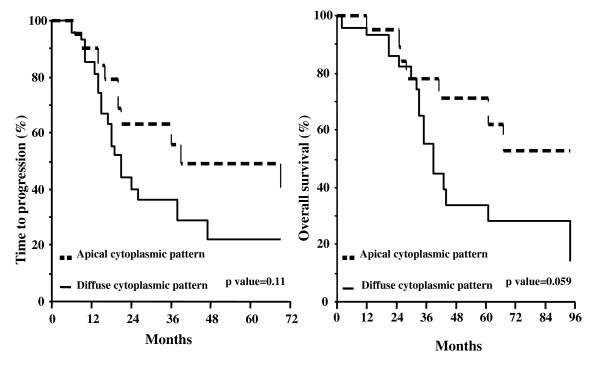
**Time to progression (A) and overall survival (B) curves in ovarian cancer patients according to the pattern of CD133 expression**.

There was no difference in the clinical outcome according to CD133 status in patients achieving primary optimal cytoreduction (p values for TTP and OS = 0.51 and 0.58, respectively), or in patients considered to be unresectable at primary laparotomy (p values for TTP and OS = 0.50 and 0.82, respectively) (data not shown). Interestingly enough, when considering the TTP and OS curves according to the pattern of CD133 expression, a trend to a better prognosis for cases with *apical cytoplasmic *versus *diffuse cytoplasmic pattern *was documented, although the statistical significance was not reached (Figure 4). We also tried to examine patients' prognosis after grouping cases with negative and *apical *cytoplasmic pattern versus cases with only *diffuse cytoplasmic pattern*. Indeed, we could not document any difference in terms of TTP (p value = 0.35) and OS (p value = 0.48) between these two groups.

In multivariate analysis of TTP and OS, advanced stage, suboptimal residual tumor at primary surgery, and response to primary treatment were found to be associated with a high risk of progression, and/or death of disease (Table 2).

**Table 2:** Multivariate analysis of clinico-pathological parameters and CD133 status as prognostic factors for TTP and OS in primary ovarian cancer patients

Variable	Multivariate for TTP		Multivariate for OS	
	
	**χ**^2^	P value	**χ**^2^	p value
**Age (yrs)**				
≤ 65				
>65	2.9	0.08	2.1	0.14

**Stage**				
I-II				
III-IV	1.7	0.2	4.1	**0.04**

**Histotype**				
Serous				
Other	0.4	0.5	0.8	0.4

**Residual tumor**				
Absent/suboptimal				
Exploratory laparotomy	18.1	**0.0001**	15.2	**0.0001**

**Response to treatment**				
No				
Yes	11.7	**0.0006**	3.4	**0.06**

**CD133 status**				
Negative				
Positive	2.5	0.11	0.1	0.9

*only variables with p value < 0.10 in the univariate analysis were included in the multivariate model

There was no difference in the clinical outcome of cases with negative versus positive CD133 expression when analyzing the subgroup of platinum resistant (TTP, p value = 0.80; OS, p value = 0.77), and platinum sensitive patients (TTP, p value = 0.85; OS, p value = 0.11) (data not shown).

## Discussion

This is the first study analyzing the immunohistochemically assessed expression of CD133 antigen in a large, single Institution series of primary ovarian carcinomas. CD133 immunoreaction appeared to be expressed at the apical/endoluminal surface of tumor cells forming a lumen, as reported in a small series of primary ovarian carcinomas [27], as well as in pancreatic [22,24], and gastric carcinoma [34], while it was mainly localized in the cytoplasm of cells forming solidly arranged tumors. CD133 positivity in the current series was in the range reported in other human solid tumors by studies employing immunohistochemical assays [12,22], confirming that only a very small proportion of tumor cells with stemness features exist within the tumor bulk.

However, the current results differ from our previous findings in that higher percentage of CD133 positive tumor cells were documented in a small series of primary ovarian carcinomas analyzed by fluorescence activated cell sorting (FACS) [26]: among the possible explanations of this discrepancy, the potential effects of mechanical and enzymatic procedures used in the preparation of cell suspension for FACS analysis should be taken into account: indeed, the disruption of the cellular microenvironment is likely to influence surface membrane antigens, although the extent of this phenomenon has been not yet addressed. In this context, a more in depth analysis of the correlation between immunohistochemically and FACS assessed CD133 positive tumor cells would be of utmost importance.

Given the strong rationale linking CD133 expression to more aggressive cellular behaviour, including resistance to chemotherapy and radiotherapy, several studies have addressed its correlation with clinico-pathological characteristics of cancer patients: in particular, a direct correlation between CD133 expression and advanced stage of disease as well as poor grade of differentiation has been shown in hepatocellular carcinoma [23]. Moreover, in pancreatic cancer the percentage of CD133 positivity correlates with metastatic lymph node involvement and also with VEGF-C expression and microvessel density [22]. As far as clinical outcome is concerned, elevated expression of CD133 has been reported to identify, even in multivariate analysis, poor prognosis patients bearing hepatocellular, pancreatic and glioma tumors [21,23].

We failed to find any association between CD133 expression and any of the clinico-pathological features examined. Moreover, we could not document any difference in terms of time to progression and overall survival according to CD133 status. These results are unlikely related to the potential bias inherent in the use of an arbitrary cut-off point, since clinical outcome was also studied utilizing the percentage of CD133 positive tumor cells as a continuous value; moreover, all patients were diagnosed, treated, and followed up at the same Institution, thus reducing the bias related to the differences in the extent of surgical cytoreduction or general management of this neoplasia. Finally, the analysis of TTP according to CD133 status minimizes the clinical impact of post-relapse chemotherapeutic regimens.

The absence of any correlation between CD133 expression and clinical outcome remains to be clarified: doubts about the limitations in the use of antibodies recognizing the AC133 and AC141 epitopes have been cast: indeed, discordant expression of the two epitopes has been documented, and it has been also demonstrated that both AC133 and AC141 can be absent despite the presence of CD133 protein [35]. In addition, it cannot be excluded that the role of CD133 as a marker of CSCs may differ according to tumor type: for instance, in gliomas and glioblastomas, CD133 expression has been shown not to be an absolute requirement for tumor initiation and progression [36,37], thus leading to hypothesize that it could just characterize populations with enhanced proliferative potential. In this context, it has to be taken into account, as emphasized by Kelly et al. [38], that the ability of CD133 positive cells to form tumors *in vivo *is also conditioned by the interaction with host microenvironment and immune system, an issue which is often ignored. Therefore, the possibility that CD133 does not represent a marker for CSCs, but rather characterizes tumor cells more effectively able to grow in other species, cannot, in principle, be ruled out. Finally, the role of the different pattern of CD133 immunoreaction has not to be underestimated: with the limits inherent in the small series of cases with positive CD133 staining, we reported that cases with *apical cytoplasmic *CD133 expression showed a trend to a better prognosis compared to cases with *diffuse cytoplasmic *pattern of immunoreaction. At present, it is unknown whether the subcellular localization of CD133 glycoprotein may affect CD133 function, or its involvement in cancer stem biology, although it has been hypothesized that the apical/endoluminal staining represents a particular step in cellular differentiation, while the less frequently represented cytoplasmic staining would more likely characterize cells with stemness features [24]. Our findings, therefore, deserve to be further investigated in a larger series.

## Conclusion

In conclusion, we showed that the immunohistochemical assessment of CD133 glycoprotein expression seems not to provide information of potential clinical value for prediction of response to treatment or prognosis in ovarian cancer patients. Uncovering the physiology, the potential tissue specificity, and the role of subcellular localization of CD133 is crucial for determining the true role of this antigen as a prognostic marker of CSCs in this neoplasia. This issue becomes even more relevant considering that attempts to target CSCs population are actively ongoing, and a chimeric molecule combining the murine anti-human CD133 and the cytotoxic drug monomethyl auristatin has been already shown to be able to inhibit the growth of gastric and hepatocellular cancer cells *in vitro *and *in vivo *[34].

## Competing interests

The authors declare that they have no competing interests.

## Authors' contributions

GF contributed to the conception and design of the study, analysis and interpretation of the results, drafting of the final manuscript. EM carried out the immunohistochemistry studies and contributed to table and figures preparation. MP contributed to the data collection and table and figures preparation. MGP carried out the immunohistochemistry studies and contributed to table and figures preparation. GFZ contributed to the histological slides evaluation, data collection and table and figures preparation. SS contributed to the histological slides evaluation, data collection and table and figures preparation. GS contributed to the conception, design of the study and interpretation of the results. All authors read and approved the final manuscript

## Pre-publication history

The pre-publication history for this paper can be accessed here:

http://www.biomedcentral.com/1471-2407/9/221/prepub
